# WHO Air Quality Guidelines 2021–Aiming for Healthier Air for all: A Joint Statement by Medical, Public Health, Scientific Societies and Patient Representative Organisations

**DOI:** 10.3389/ijph.2021.1604465

**Published:** 2021-09-23

**Authors:** Barbara Hoffmann, Hanna Boogaard, Audrey de Nazelle, Zorana J. Andersen, Michael Abramson, Michael Brauer, Bert Brunekreef, Francesco Forastiere, Wei Huang, Haidong Kan, Joel D. Kaufman, Klea Katsouyanni, Michal Krzyzanowski, Nino Kuenzli, Francine Laden, Mark Nieuwenhuijsen, Adetoun Mustapha, Pippa Powell, Mary Rice, Aina Roca-Barceló, Charlotte J. Roscoe, Agnes Soares, Kurt Straif, George Thurston

**Affiliations:** ^1^ Institute for Occupational, Social and Environmental Medicine, Medical School, Heinrich-Heine-University of Düsseldorf, Düsseldorf, Germany; ^2^ Health Effects Institute, Boston, MA, United States; ^3^ Imperial College London, London, United Kingdom; ^4^ Department of Public Health, University of Copenhagen, Copenhagen, Denmark; ^5^ School of Public Health and Preventive Medicine, Monash University, Melbourne, VIC, Australia; ^6^ School of Population and Public Health, University of British Columbia, Vancouver, BC, Canada; ^7^ Institute for Risk Assessment Sciences, Utrecht University, Utrecht, Netherlands; ^8^ Department of Occupational and Environmental Health, Peking University, Beijing, China; ^9^ School of Public Health, Fudan University, Shanghai, China; ^10^ Department of Environmental and Occupational Health Sciences, School of Public Health, University of Washington, Seattle, WA, United States; ^11^ Department of Hygiene, Epidemiology and Medical Statistics, Medical School, National and Kapodistrian University of Athens, Athens, Greece; ^12^ Swiss Tropical and Public Health Institute (Swiss TPH), Basel, Switzerland; ^13^ Harvard T.H. Chan School of Public Health, Boston, MA, United States; ^14^ Instituto Salud Global Barcelona (ISGlobal), Barcelona, Spain; ^15^ Nigerian Institute of Medical Research, Yaba, Lagos, Nigeria; ^16^ European Lung Foundation, Sheffield, United Kingdom; ^17^ Pan American Health Organization, Washington D.C., DC, United States; ^18^ Boston College, Chestnut Hill, MA, United States; ^19^ Department of Population Health, New York University School of Medicine, New York City, NY, United States

**Keywords:** air pollution, WHO Air Quality Guidelines, health effects, policy implications, average population exposure

After years of intensive research and deliberations with experts across the globe, the World Health Organization (WHO) updated its 2005 Global Air Quality Guidelines (AQG) in September 2021 [1, 2]. The new air quality guidelines (WHO AQG) are ambitious and reflect the large impact that air pollution has on global health. They recommend aiming for annual mean concentrations of PM_2.5_ not exceeding 5 µg/m^3^ and NO_2_ not exceeding 10 µg/m^3^, and the peak season mean 8-hr ozone concentration not exceeding 60 µg/m^3^ [1]. For reference, the corresponding 2005 WHO guideline values for PM_2.5_ and NO_2_ were, respectively, 10 µg/m^3^ and 40 µg/m^3^ with no recommendation issued for long-term ozone concentrations [3]. While the guidelines are not legally binding, we hope they will influence air quality policy across the globe for many years to come.

The updated WHO AQGs have become necessary as an overwhelming body of evidence has accumulated over the past two decades, demonstrating that health effects of air pollution are serious and can affect nearly all organ systems of the human body [4]. Importantly, recent studies and large research programmes consistently show that the adverse effects of air pollution are not only limited to high exposures; harmful health effects can be observed all the way down to very low concentration levels, with no observable thresholds below which exposure can be considered safe [5–7].

There is now broad expert consensus that air pollution is a major global public health risk factor and puts an enormous financial burden on societies. Outdoor and household air pollution together accounted for approximately 12% of all deaths in 2019. Air pollution currently ranks fourth among major risk factors for global disease and mortality, only behind hypertension, smoking and dietary factors [8]. In terms of economic burden, the estimated global health-related external costs (i.e., those borne by society as a whole) were US$ 5 trillion in 2013 with an additional US$ 225 billion in lost labour productivity [9]. For the WHO European Region, the overall annual economic cost of health impacts and mortality from air pollution, including estimates for morbidity costs, stood at US$ 1.575 trillion [10].

The most important message of the updated WHO AQG is that each reduction in the outdoor concentrations of key air pollutants brings health benefits to the surrounding population, even in places which already have low pollution concentrations. Moreover, linear exposure-response relationships down to the lowest observable concentrations show that every individual will benefit from cleaner air [11–15]. These findings provide critical input into clean air policies and regulation around the world. They also are key to estimating the potential health and economic benefits from policies that reduce exposure to air pollution.

Recognising that the adverse health effects of pollution exposure can be seen at all, even at the lowest, observed levels of pollution concentrations, is a milestone for cleaner air and better health policies; it offers a wake-up call, to reconsider current air quality legislation and regulations. To maximise health benefits, we now understand better the importance of implementing measures to reduce average exposures of all people. Such an approach must complement reductions in exposure at “hotspots” with high levels of air pollution, in particular to address known inequities owing to socioeconomic conditions, increased vulnerability of the residential population, and economic activities [16]. To tackle the health effects of air pollution, bold air quality actions are needed at all levels–international, national, local–and across all sectors such as transport, energy, industry, agriculture, and residential.

Most jurisdictions with clean air regulations have relied on fixed limit values with little incentive to further reduce air pollution levels once compliance with the limit value is achieved [17]. Given the evidence that health effects occur all the way down to very low concentration levels, future clean air policies must include incentives for progressive lowering of exposures of the entire population, thereby improving health for all. What is needed is a paradigm change from relying solely on fixed limit values, with a shift towards the concept of combining fixed limit values with a continuous reduction of the average exposure. For example, the current European Union (EU) Ambient Air Quality Directive already contains a non-binding average exposure reduction target [18]. The upcoming 2022 revision of the EU Ambient Air Quality Directive will offer the chance to lead the way and implement binding average exposure reduction goals for air pollutants in combination with lowered fixed limit values.

Programmes that reduce air pollutant emissions provide enormous air quality and health benefits which increase over time. The estimated health benefits of improved air quality outweigh by far the implementation costs of air quality actions. For the US, it has been estimated that the benefits from decreased mortality, lower medical expenditures for air pollution-related diseases, and higher productivity of workers are around 30 times greater than the costs of the Clean Air Act, resulting in net improvements of economic growth and population welfare [19]. In China, public health benefits were 50% greater than the costs for air quality improvement measures [20]. Similarly, for the EU, additional clean air and climate policies beyond the current obligations will lead to net benefits with positive macro-economic implications [21]. Indeed, the cost effectiveness of air quality actions is enhanced by the close link between air pollution and greenhouse gas emissions. A reduction of air pollution emissions will also feed into efforts for climate neutrality and vice versa, making benefits from investments in one area count twice [22, 23].

## Conclusion

Air pollution is a major global public health threat that causes a range of adverse health effects, even at the lowest observable concentrations. There is ample evidence to strongly support government action to reduce air pollution and address climate change simultaneously. The updated WHO AQG are bold and stress the importance of lowering air pollution concentrations at every level. The benefits are clear: lowering air pollution levels will lead to enormous improvements in public health for people of all ages breathing cleaner air. We support the recommendations of the new WHO AQG, and urge nations to use the WHO AQG as a guide for ambitious air quality and emission reduction policies around the world.

The initiative for the Joint Society Statement was led by the European Respiratory Society (ERS) and the International Society of Environmental Epidemiology (ISEE).

The Joint Society Statement was endorsed by

• Alpha 1 España
• Alpha 1 Germany
• Alpha-1 Belgium
• American Cancer Society
• American Heart Association
• American Lung Association
• American Public Health Association (APHA) Epi section
• American Thoracic Society
• APH Macedonia “Moment plus”
• Asian Pacific Society of Respirology
• Asociación Argentina de Medicina Respiratoria
• Asociación de Apoyo e Información a Familiares y Pacientes con Neumonía (NEUMOAI)
• Asociación de Pacientes con EPOC (APEPOC)
• Asociación Nacional de Hipertensión Pulmonar
• Aspergillosis Trust
• Association for Respiratory Technology & Physiology (ARTP)
• Association of Physicians of India
• Association of Schools of Public Health in the European Region (ASPHER)
• Associazione Culturale Pediatri
• Associazione Italiana Bronchiettasie
• Associazione Italiana di Epidemiologia
• Associazione Italiana Ictus
• Associazione Nazionale Alfa 1
• Associazione Nazionale Alfa 1-At ODV
• Asthma UK ‐ British Lung Foundation
• Belgian Respiratory Society
• Breathe Easy
• Bulgarian Society of the Patients with Pulmonary Hypertension (BSPPH)
• Canadian Lung Association
• Canadian Society for Epidemiology and Biostatistics (CSEB)
• Cardiological Society of India
• Center for Indonesian Medical Students’ Activities (CIMSA) CF Europe
• Collegium Ramazzini
• Deutsche Gesellschaft für Kardiologie (DGK)
• Dutch Heart Foundation
• Ethiopian Thoracic Society
• European Alliance of Associations for Rheumatology
• European Cancer Organisation (ECO)
• European Cancer Patient Coalition (ECPC)
• European Chronic Disease Alliance
• European Federation of Allergy and Airways Diseases Patients’ Associations
• European Lung Foundation (ELF)
• European Public Health Alliance
• European Pulmonary Fibrosis Federation (EU-IPFF)
• European Society of Cardiology (ESC)
• European Society of Thoracic Surgeons
• Fair Life
• Federasma Allergie
• FENEAR
• Foundation for Sarcoidosis Research
• French Society of Respiratory Diseases (SPLF)
• German Association for Medical Informatics, Biometry and Epidemiology (GMDS)
• German Society for Epidemiology (DGEpi)
• German Society for for Social Medicine and Prevention (DGSMP)
• German Society for for Social Medicine and Prevention (DGSMP)
• German Society for Public Health (DGPH)
• Gesellschaft für Pädiatrische Pneumologie
• Healthy Lungs-Nepal
• Helenic Thoracic Society
• Helping Hands Foundation
• Hypertention, Infaction & Stroke Prevention Association
• Ibero-American Environmental Health Society
• Inspire
• International Commission on Occupational Health (ICOH)
• International Network for Epidemiology in Policy (INEP)
• International Society for Aerolols in Medicine
• International Society of Environmental Epidemiology
• International Union Against Tuberculosis and lung disease
• Italian Association of Allergologists and Immunologists
• Italian Thoracic Society-Italian Association of Hospital Pulmonologists
• Korean Academy of Medical Sciences
• Korean Academy of Tuberculosis and Respiratory Diseases
• Korean Society of Epidemiology
• Korean Society for Preventive Medicine
• Korean Society of Occupational and Environmental Medicine
• LAM Action
• Lebanese Pediatric Society
• LongFonds
• Lovexair
• Lung Cancer Europe
• Malaysian Thoracic Society
• National Association of County Health Officials (NACCHO)
• Netherlands Respiratory Society
• Patiëntenvereniging longtransplantatie UZ Leuve (HALO vzw)
• PHA Europe
• Philippine College of Chest Physicians
• Polskie Towarzystwo Chorób Płuc
• Pulmonary Fibrosis Trust
• Research group in statistics and econometrics in Public Health
• Rete Italiana Ambiente e Salute
• Sarcoidose.nl
• Sarcoidosis UK
• Sociedad Española de Neumología y Cirugía Torácica (SEPAR)
• Sociedade Brasileira de Pneumonologia e Tisiologia
• Società Italiana Allergologia, Asma ed Immunologia Clinica
• Società Italiana di Pediatria
• Società Italiana di Pneumologia
• Società Italiana per le Malattie Respiratorie Infantili
• South African Thoracic Society
• Stichting Huize Aarde
• Swedish Asthma and Allergy Association
• Swedish Heart-Lung Foundation
• Swiss Society of Pneumology
• Taiwan Society of Pulmonary and Critical Care Medicine
• The Thoracic Society of Australia & New Zealand
• Turkish Respiratory Society
• Turkish Thoracic Society
• Union for International Cancer Control’s (UICC)
• World Heart Federation
